# Radiobiological Aspects of FLASH Radiotherapy

**DOI:** 10.3390/biom12101376

**Published:** 2022-09-26

**Authors:** Eline Hageman, Pei-Pei Che, Max Dahele, Ben J. Slotman, Peter Sminia

**Affiliations:** 1 Amsterdam UMC Location Vrije Universiteit Amsterdam, Radiation Oncology, Boelelaan 1117, 1081 HV Amsterdam, The Netherlands; 2 Cancer Center Amsterdam, Cancer Biology and Immunology, 1081 HV Amsterdam, The Netherlands

**Keywords:** FLASH, radiobiology, radiotherapy, ultra-high dose rate, healthy tissue sparing, tumor control

## Abstract

Radiotherapy (RT) is one of the primary treatment modalities for cancer patients. The clinical use of RT requires a balance to be struck between tumor effect and the risk of toxicity. Sparing normal tissue is the cornerstone of reducing toxicity. Advances in physical targeting and dose-shaping technology have helped to achieve this. FLASH RT is a promising, novel treatment technique that seeks to exploit a potential normal tissue-sparing effect of ultra-high dose rate irradiation. A significant body of in vitro and in vivo data has highlighted a decrease in acute and late radiation toxicities, while preserving the radiation effect in tumor cells. The underlying biological mechanisms of FLASH RT, however, remain unclear. Three main mechanisms have been hypothesized to account for this differential FLASH RT effect between the tumor and healthy tissue: the oxygen depletion, the DNA damage, and the immune-mediated hypothesis. These hypotheses and molecular mechanisms have been evaluated both in vitro and in vivo. Furthermore, the effect of ultra-high dose rate radiation with extremely short delivery times on the dynamic tumor microenvironment involving circulating blood cells and immune cells in humans is essentially unknown. Therefore, while there is great interest in FLASH RT as a means of targeting tumors with the promise of an increased therapeutic ratio, evidence of a generalized FLASH effect in humans and data to show that FLASH in humans is safe and at least effective against tumors as standard photon RT is currently lacking. FLASH RT needs further preclinical investigation and well-designed in-human studies before it can be introduced into clinical practice.

## 1. Introduction

External beam radiotherapy (RT) is one of the most important treatment modalities for the cure and palliation of cancer. Around 50% of cancer patients are treated with RT alone or in combination with other treatment modalities, such as chemotherapy [1]. However, the quality of life of cancer patients can be impacted by short- and long-term adverse effects due to radiation exposure of surrounding healthy tissue while delivering the prescribed RT dose to the tumor that is typically situated deep in the patient [2]. Hence, the essence of RT is effectively killing tumor cells with minimal exposure of the neighboring healthy tissues. This can be achieved in two main ways: (1) precise deposition of ionizing radiation (IR) energy to the tumor site only with limited exposure of surrounding healthy tissue, and (2) differential biological radiation response between tumor and healthy tissue. Over the past decades, advances in technology have improved precision delivery of radiation with high conformity to the target. With conventional radiotherapy (CONV)—typically using 2 Gy fractions/day for five days over several weeks—discrimination between tumor and normal tissue responses is achieved by the tissue-sparing effect of dose fractionation, allowing healthy tissues to recover to a greater extent than the tumor [3]. However, in the treatment of radio-resistant tumors such as high-grade brain and pancreatic malignancies, the total radiation dose that can be delivered to the tumor is often still limited by the radiation tolerance of neighboring, critical, normal tissues. In such situations, ultra-high dose rate FLASH RT could be of significant interest.

**Figure 1:**
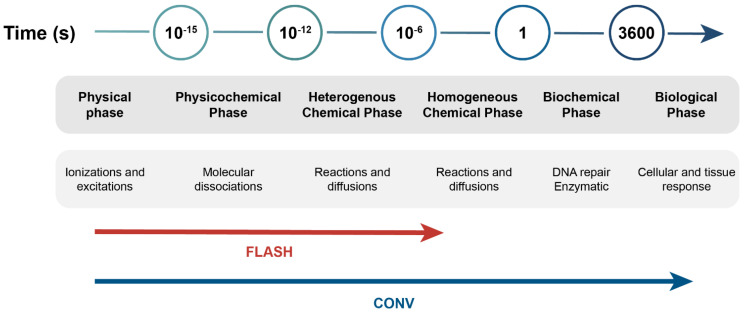
Schematic overview of early physical, chemical, and biological phases following radiation exposure of cells and tissues. CONV interferes with the chemical and biological steps, while FLASH does not interact with the biochemical steps. Adapted from Vozenin et al. (2019) [5].

The FLASH RT strategy is typically considered to comprise radiation dose delivery to the target volume at a dose rate of ≥40 Gy/s, whereas CONV uses dose rates of 0.01–0.1 Gy/s [3]. Due to different delivery times, CONV irradiation takes place during chemical and biological responses, whereas FLASH does not interact with these biochemical steps (Figure 1) [4,5].

The history of FLASH dates back to at least 1966. Following whole body exposure, the survival of mice treated at a dose rate between 0.20 and 500 Gy/min was investigated [6]. The observations showed a decrease in survival with increasing dose rate, however reaching a plateau and even a slight increase in survival at dose rates exceeding 100 Gy/min. Interestingly, the authors conclude that “it could be expected that at very high dose-rates local oxygen depletion would start to play a part in reducing the effectiveness of radiation”. This early observation of the very high dose rate sparing effect was also demonstrated in the 1970s for intestine and skin [7,8,9]. The potential clinical exploitation of the dose rate effect was discussed, considering such factors as normal and tumor cell death, the role of oxygen tension, and radiation resistance. Eventually, these early experimental findings were not translated into the clinic; it was considered that the total dose required to consume all the oxygen in oxic cells would be too high and not clinically achievable [6]. Studies suggested that 5–10 Gy at a rate of 10^9^ Gy/s was required to deplete cellular oxygen, significantly changing the radiosensitivity of low oxygen tension cells [10]. In addition, studies focusing on tumor control were lacking [11]. Nearly 30 years later, ultra-high dose rate effects are being “rediscovered”, and these are nowadays often referred to as FLASH RT, typically using a dose rate of >40 Gy/s. Current experimental data generally indicate that FLASH has a sparing effect on healthy tissue by decreasing the acute and even late toxicities while maintaining the same tumor control probability as with CONV dose rates [4]. Healthy tissue sparing has been demonstrated in several mice studies for multiple organ systems, such as lung, brain, intestinal tract, and skin [12,13,14,15]. The sparing effect has also been seen in larger animals such as a mini pig, cats, dogs and the first human patient has been treated [16,17].

**Figure 2:**
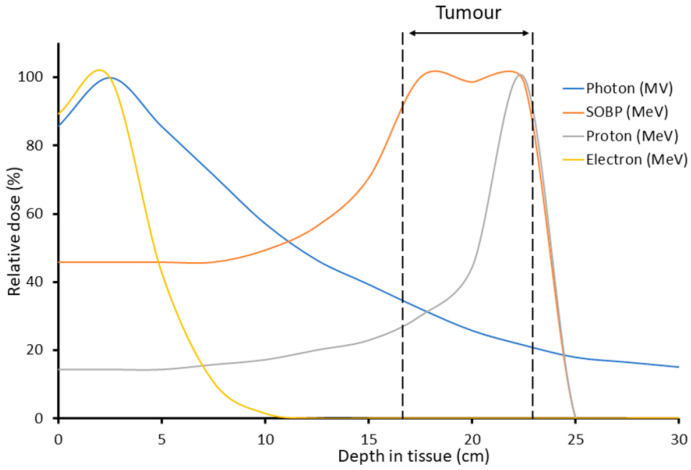
Depth–dose distribution between different sources of IR. Differences in entry dose and Bragg peak—delineated in dashed black lines—between the different radiation modalities are highlighted. Proton beam treatment allows for more precise targeting, due to its low entry dose, with the majority of the beam’s energy delivered in the Bragg peak. Reproduced with permission from Hughes and Parsons, *Int. J. Mol. Sci.*, 2020 [2]. Abbreviations: SOBP = spread-out Bragg peak.

At present, studies regarding the biological mechanisms of FLASH RT are incomplete and far from conclusive [3]. Here, we discuss putative biological mechanisms. Along with these biological mechanisms, physical factors of IR delivery involved in the FLASH effect are considered, including total dose, pulse rate/duration/width/number, and total delivery time [2]. Many of the current FLASH investigations use electron linear accelerators [18,19,20,21]. However, electron beams are limited to the treatment of superficial tumors due to the low tissue penetration and limited field size of the beams. Proton beam therapy (PBT) is currently seen as the most promising for clinical application, as it offers the greater tissue penetration depth and therefore allows irradiation of deep-seated tumors. Another important advantage of PBT over CONV photon RT is the lower entrance dose. Furthermore, the majority of the beam energy is deposited in the characteristic Bragg peak, which further increases the preferential targeting of the tumor volume and reduces the exposure of healthy tissues (Figure 2). For those reasons, proton FLASH is currently of considerable interest. However, there are some biological uncertainties around PBT, in particular regarding the possible increase in the linear energy transfer (LET) around the Bragg peak, which could lead to changes in the DNA damage spectrum and increases in relative biological effectiveness [2].

## 2. Biological Mechanisms

The biological mechanisms responsible for the reduction of normal tissue toxicities with FLASH irradiation compared to CONV are not fully understood. However, there are some non-mutually exclusive hypotheses that have been proposed. These are summarized in Table 1 and discussed in further detail below. Most FLASH preclinical studies have used electron/photon irradiation, and a few studies have used protons. Of importance is that high-energy (low-LET) protons are thought to generate a DNA damage spectrum similar to that of X-rays and γ-irradiation, whereas low-energy protons (with increased LET), specifically at the Bragg peak distal end (Figure 2), generate complex DNA lesions with increasing frequency, which are difficult to repair [22]. Note that studies performed with proton FLASH are generally compared with photon CONV as a control; for the reason that there is no development in proton CONV. However, because of the small high-LET component of proton exposure, the overall biological effects on both malignant and normal tissues between photon FLASH and proton FLASH irradiation are not expected to be very different. Future proton FLASH studies will certainly address the biological mechanism issue [22].

### 2.1. Oxygen Depletion/ROS

**Figure 3:**
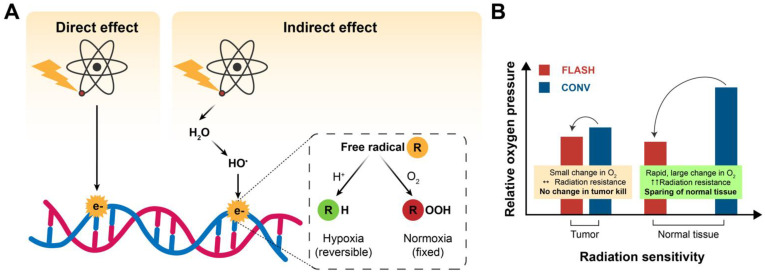
The FLASH effect as explained by the oxygen depletion hypothesis. (**A**) Indirect DNA damage occurs through radiolysis of water and generation of hydroxyl radicals, which can be easily resolved. When this radical reacts with molecular oxygen, a peroxyl radical is created, and the DNA damage becomes fixed. Adapted from Grimes et al. (2015) [12]. (**B**) FLASH RT causes rapid depletion of oxygen, hereby the healthy tissue is spared while the tumor control is maintained. The change in relative oxygen pressure and radiation sensitivity between FLASH and CONV RT for tumor and normal tissue are depicted with arrows. Adapted from Wilson et al. (2020) [34].

Hypoxic tissues are more radio-resistant than well-oxygenated cells due to the absence of molecular oxygen, which causes fixation of indirect radiation-induced DNA damage [34]. The oxygen fixation hypothesis suggests that in response to IR, indirect DNA damage occurs via radiolysis of water and generation of reactive oxygen species (ROS), such as hydroxyl radicals. These free radicals can incorporate into the DNA, causing damage that can be easily resolved. However, when these free radicals react with molecular oxygen, a peroxyl radical is created, resulting in a DNA lesion that is difficult to repair (Figure 3A). It is well known that a lack of oxygen in the immediate environment can limit radiation-induced DNA damage [2,34,35]. For low-LET radiation, 60–70% of the indirect radiation damage is induced by ROS. The super-fast delivery of a single dose of FLASH RT can increase the resistance of healthy tissue to IR by depletion of oxygen in normal tissue cells [2,3]. Oxygen depletion will have less effect on oxygen-deprived tumor tissue due to the presence of abnormal blood vessels and adaptation to abnormal oxygen supplementation. In terms of killing tumor cells, for the same dose of CONV and FLASH RT, the outcome would be similar. Radio-resistance in the healthy tissue and not in the tumor tissue lies at the heart of the intrinsic difference in their response to ROS for the following reasons: (1) FLASH converts local oxygen in the tissue into organic hydroperoxides, and (2) differences in capillary oxygen tension [24]. Normal tissue possesses a greater reserve capacity for the enzymatic reduction of hydroperoxides and can therefore remove them more rapidly [25,36]. Next, the capillary oxygen tension is higher in healthy tissues, which is important for regulating redox homeostasis by mitochondrial respiration and endogenous ROS [24].

**Figure 4:**
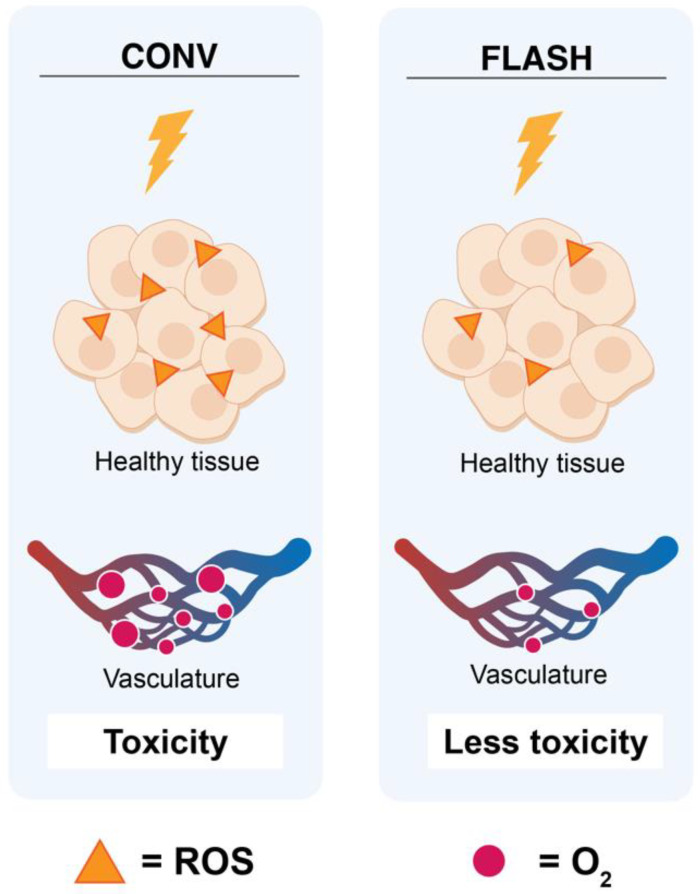
The FLASH oxygen hypothesis. FLASH RT causes rapid depletion of oxygen. Therefore, the induction of ROS might be reduced, and consequentially healthy tissue toxicity might also be reduced. Adapted from Montay-Gruel et al. (2019) [4].

The larger the difference in oxygen levels between healthy and tumor tissue, the better the differential response to FLASH (Figure 3B) [24]. To illustrate the role of oxygen depletion with the FLASH effect, Adrian et al. (2020) compared FLASH RT (600 Gy/s) and CONV (14 Gy/s) on prostate cancer cells under various oxygen concentrations [23]. Colony formation assays were used to determine the survival after exposure to doses up to 25 Gy. The results showed no difference between FLASH and CONV under normoxic conditions and hypoxia up to 5–10 Gy. However, starting from 15 Gy, FLASH showed an increase in cell survival, dependent on the oxygen concentration, with significant cell survival at 18 Gy [23]. Clonogenic cell survival data following FLASH and CONV irradiation with carbon ions of hamster ovary cells demonstrated an oxygen-dependent sparing effect of FLASH at 0.5%–4% O_2_ [37]. Khan et al., studied the effects of FLASH on oxygenation in multicellular tumor spheroids [38]. They showed that upon FLASH irradiation, the hypoxic core transiently expanded, engulfing a large number of well-oxygenated cells. In contrast, oxygen was steadily replenished during slower CONV irradiation. FLASH radiation led to a three-fold higher clonogenic survival than CONV, and a modifying factor of 1.3 above 10 Gy. Their data confirm that oxygen depletion could be an important part of the FLASH effect. However, the sparing effect of FLASH irradiation in their in vitro tumor model did not match with most in vivo data, showing similar results to the tumor control (Table 2). This might be due to the fact that spheroids lack the vasculature and are not subjected to immunogenic cell death. Hence, the tumor microenvironment could be of particular interest for FLASH [38]. In addition, the hypothesis of oxygen depletion is supported by Montay-Gruel et al. [4] (2019). In their experimental study, they doubled—via carbogen breathing—the presence of oxygen in the brain of healthy mice irradiated with FLASH, losing the protective effects of FLASH as a consequence [4]. While the oxygen depletion theory could explain the relative sparing of normal tissues to radiation (Figure 4), it does not adequately explain how FLASH can maintain tumor control compared to CONV, since most tumors show hypoxic niches [34]. The oxygen depletion hypothesis has recently been challenged. Via direct measurements of tissue pO_2_ values in normal murine tissues, it was demonstrated that pO_2_ values deceased after FLASH RT compared with CONV, but the effect was small and likely insufficient to produce hypoxia [39]. In another study, oxygen consumption was measured in sealed, 3D-printed water phantoms during irradiation with X-rays, protons, and carbon ions at varying dose rates up to 340 Gy/s. It was demonstrated that FLASH irradiation consumed oxygen, but not enough to deplete all the oxygen present [40]. Spitz et al. [25,36] showed higher levels of redox active iron (labile iron) in tumors than in normal tissues, and hence a difference in their oxidative metabolism. The authors proposed that FLASH seeds a much greater amount of hydroperoxides into tissue than CONV, and that normal cells have a greater capacity to eliminate peroxidized compounds compared to tumors [25,36]. This differential ROS-damage recovery hypothesis describes that normal and tumor cells have different capabilities to “detoxify” themselves from ROS [41]. Another explanation might be found in the kinetics of ROS. From studies using a physicochemical model, Labarbe et al. (2020) demonstrated that radical recombination shortens the lifetime or limits the radiolytic yield of organic peroxyl radicals and therewith likely protects normoxic tissues against the deleterious effects of FLASH radiation [42].

### 2.2. DNA Damage

The classic target theory considers DNA as the major target of IR. As a lethal effect of radiation, unrepaired DNA double strand breaks (DSBs) are considered to determine the fate of the cell [3,43]. In FLASH, the intrinsic factors “yield of DNA damage” and “clustered DNA damage” is likely involved in the differential response between healthy and tumor tissue [3].

Using yH2AX as a marker of DNA DSBs, FLASH was shown to generate smaller amounts of DSBs compared to CONV [26]. Less DNA damage and sparing of lung progenitor cells was observed with FLASH irradiation compared to CONV [12]. FLASH reduced the number of senescence cells in both normal lung fibroblasts and lung progenitor cells [12,26]. In addition, ultra-high dose rates of FLASH might induce more clustered DNA damage [3,27,44]. The difference between healthy and tumor tissue in response to clustered DNA damage remains unclear; it might be attributed to activation of different factors involved in DNA repair pathways or the immune system. Within these processes, poly (ADP-ribose) polymerases (PARPs) play a multifunctional role. PARP1 downregulates TGF-β, which regulates the radiation-induced anti-tumor response, while PARP2 affects the repair of pro-apoptotic DNA damage [3,28,29]. Lastly, other factors are induced by DNA damage as well. FLASH RT can lead to the massive induction of cytosolic DNA, which initiates the cGAS-STING pathway. This pathway induces the expression of interferon and other innate immune factors to promote senescence, cell death, or tissue injury, such as fibrosis [3,30]. Hence, differential activation of the cGAS-STING pathway between normal and tumor cells [31] might play a role in the FLASH effect regarding tumor control and sparing tissue from injuries such as fibrosis [3].

### 2.3. Immune Response

Inflammatory and immune responses might further contribute to the FLASH effect [2,3,34]. Intrinsic factors might change the expression and activation of immune factors and immune cells or indirectly influence immunoreaction upon induction of DNA damage or disturbance of the surrounding microenvironment of exposed tissue [3]. An important intrinsic factor is TGF-β, which mediates radiation-induced anti-tumor responses and regulates the production of ROS and DNA repair [2,3]. Whereas the activation of the TGF-β pathway is observed in CONV, FLASH possibly avoids the induction of this pathway, resulting in a decrease of ROS and DNA damage; however, this remains to be evaluated. Additionally, the differences between FLASH and CONV regarding the activation and localization of T cells still needs to be elucidated. However, an increase in T-lymphocyte recruitment into the tumor microenvironment of lung tumor-bearing mice irradiated with FLASH has been reported, compared to CONV [32]. While CONV leads to an increase in presenting tumor antigen, and cytokine release, resulting in immunogenic cell death and modulation of immunogenicity, these processes might be different for FLASH [3]. Indeed, FLASH irradiated Lewis lung carcinoma cells induced changes in the tumor microenvironment, such as decreased phosphorylated myelin light chain activation, increased CD31+ endothelial cell area density, as well as an increased number of γH2AX (DNA DSB marker) positive cells [45]. Furthermore, they demonstrated an increase in immune cell infiltration of T cells and myeloid cells into the tumors after FLASH RT.

## 3. In Vivo Studies

Over the last several years, a number of in vivo studies has been published on the effect of FLASH-RT compared with CONV. Studies were performed on various normal tissues to determine acute and late effects, as well as on efficacy regarding tumor control. In general, single doses were studied, with only a few studies applying multiple fractions. Different radiation sources were used. Studies are described below and listed in Table 2.

### 3.1. Mice Brain

When whole mice brains were irradiated with 10 Gy using electrons at FLASH dose rates, long-lasting preservation of cognitive memory skills was observed starting from a mean dose rate of 30 Gy/s and increasing up to 100 Gy/s, while CONV induced irreversible alteration in memory [4,19,46,47]. Similarly, the same sparing effect could be observed for X-rays at a mean dose rate of 37 Gy/s [13]. However, this sparing FLASH effect on neurocognitive functions in nude mice was already diminished after 14 Gy, but in hypofractionation schemes of 2 × 7 Gy and 3 × 10 Gy, mice retained similar neurologic function in recognition memory as non-irradiated groups [46]. Preservation of cellular division in the hippocampus after FLASH irradiation might be associated with the relative preservation of neurogenesis and glial cell production in this memory-involved brain region [13]. Moreover, FLASH induced less reactive astrogliosis in the irradiated brain, which is highly involved in brain homeostasis. This reduced toxicity is consistent with preservation of cognitive functions and hippocampal cell division. As a result, neuroinflammation appeared to be a differential effect between CONV RT and FLASH RT, as CONV RT increased CD68-positive microglia activation [4,14]. Reduced levels of ROS seem to be involved in the FLASH effect after whole brain irradiation in mice. However, doubling the oxygen pressure eliminated the neurocognitive preservation with FLASH [4]. Furthermore, CONV RT induced an increase in 5 out of a panel of 10 pro-inflammatory cytokines in the hippocampus, while FLASH was associated with a smaller increase in only 3 cytokines, highlighting the involvement of the immune system induced by radiation. Interestingly, 2 × 8 Gy in subcutaneously injected glioblastoma cells in immunocompetent mice resulted in long-term anti-tumor efficacy up to 100 days, after CONV and FLASH RT [48]. After mice were cured from their tumors, these anti-tumor effects persisted even after a second inoculation with these cells on the contralateral side. However, in an intracranial setting, anti-tumor efficacy was less pronounced, despite raising the dose scheme to 2 × 12.5 Gy, possibly indicating the involvement of the tumor microenvironment (TME) or the blood–brain barrier.

### 3.2. Mice Abdomen

Several studies compared CONV and FLASH RT after whole or focal abdomen including tumor [49,50,51]. In a study to investigate acute intestinal toxicity, whole abdomen and focal tumor-bearing abdomen in mice were irradiated to compare CONV with FLASH RT [49]. While whole abdominal irradiation of 15 Gy at both CONV and FLASH dose rates significantly reduced proliferating cells per crypt, proton FLASH RT, however, spared more compared to CONV [49]. This resulted in a significant increase in regenerated crypts. Moreover, focal abdominal radiation of 18 Gy with FLASH resulted in less pronounced fibrotic development compared to CONV. However, tumor growth control was similar for both CONV and FLASH RT [49].

Similarly, Levy et al. (2019) compared FLASH (216 Gy/s) and CONV (0.079 Gy/s) whole abdominal irradiation in normal mice and mice with ovarian cancer to investigate radiation-induced gastrointestinal toxicity associated with total abdominal irradiation as an adjuvant treatment in metastatic ovarian cancer [50]. They established that FLASH resulted in less lethality from radiation-induced gastrointestinal syndrome than CONV. Both groups of mice irradiated with 16 Gy CONV or FLASH lost more than 25% of their body weight, but 90% recovered and survived only within the FLASH group. In the normal mice, no difference was found in hematopoietic toxicity, and all mice expressed mucosal damage. However, mice irradiated with FLASH had a two-fold increase in regenerating crypts, and the intestinal mucosa of the surviving mice was indistinguishable from non-irradiated mice. Furthermore, they demonstrated that a sub-lethal dose of 14 Gy FLASH induced less apoptosis in crypt base columnar cells and less early DNA damage and therewith better spared the intestinal function and epithelial integrity than CONV [50].

To further elaborate on the FLASH-sparing effect on normal tissue, Ruan et al. investigated the effect of different temporal pulses and dose rates compared to CONV RT [51]. Within their murine models, sparing of the gastrointestinal function at FLASH dose rates was found for doses delivered between 7.5 and 12.5 Gy. They observed that a single pulse at the highest dose rate resulted in the most optimal sparing of the intestinal crypt, similar to 7–8 pulses at an average dose rate of 216 Gy/s [50,51]. Interestingly, diversity in gut microbiota was also differentially affected after radiation. After CONV RT decreased richness could be observed and was positively associated with increased intestinal injury [51].

### 3.3. Mice Lungs

Lung xenografts in mice were irradiated with CONV (<0.03 Gy/s) versus FLASH (>40 Gy/s) [18]. With respect to the early effects of FLASH RT, it was demonstrated that FLASH RT protects blood vessels and bronchi from radiation-induced apoptosis. Regarding long-term effects, FLASH RT decreased radiation-induced lung fibrosis. FLASH was as efficient as CONV in controlling xenografted human tumors and syngeneic orthotopic lung tumors.

FLASH RT, relative to CONV, showed less DNA damage and death in normal human lung cells in vitro [12]. Following FLASH RT of murine lungs, FLASH reduced the pressure to repopulate cells after radiation injury, minimized the induction of pro-inflammatory genes and reduced the proliferation rate of progenitor cells after injury. In late stages, FLASH was associated with less persistent DNA damage and fewer senescent cells than after CONV exposure, suggesting a higher potential for lung regeneration after FLASH RT. One out of eight wild-type (WT) mice irradiated with FLASH developed fibrosis compared to 10 of 11 WT mice irradiated with CONV. In Terc−/− mice (telomerase negative), the FLASH effect seemed to be lost, since almost all mice showed signs of fibrosis [12].

Interestingly, murine lung carcinomas treated with 18 Gy proton FLASH irradiation were significantly smaller than after CONV [32]. FLASH increased recruitment of CD3+ T lymphocytes from the peripheral tumor edge into the tumor core, and both CD4+ and CD8+ cells were also increased in the core, which might account for the high tumor control for mice irradiated with FLASH.

### 3.4. Anti-Tumor Efficacy in Mice

Mice with lymphoblastic leukemia and normal hematopoiesis were irradiated with 4 Gy FLASH RT at 200 Gy/s versus 4 Gy CONV at less than 0.07 Gy/s [52]. Evaluation of the long-term effects of FLASH in two patient-derived xenografts (PDX) showed a larger decrease in leukemic cells compared to CONV, and this tumor control was maintained up to 7 weeks only for FLASH-treated mice. Moreover, a stronger inhibitory effect of FLASH on the growth potential of T cell acute leukemic cells was seen. However, in the other two PDXs, FLASH induced a delayed progression, while CONV cured both mice. This suggests that individual intrinsic factors are able to differentially drive the response of human T cell acute leukemic cells. Further assessment of underlying genetic factors showed that the two FLASH-sensitive cases had similar genetic abnormalities and a presumed susceptibility imprint to FLASH RT. Lastly, this study demonstrated the preservation of hematopoietic stem/progenitor cells after FLASH RT. Moreover, FLASH could control tumor development in three out of four cases, whereas CONV-treated cells died from leukemia infiltration [52].

In both mouse models with ovarian cancer peritoneal metastasis and pancreatic flank tumors, FLASH, using protons or electrons, had a similar tumor control efficacy compared to CONV, but produced less intestinal injury [50,51]. Taken together, these results underline the sparing of normal tissue only at FLASH dose rates.

### 3.5. In Vivo Mice Studies with Negative Results for FLASH

While most studies of FLASH RT have shown positive results, the study from Venkatesulu et al. did not [53]. The authors evaluated early effects in mice with lymphopenia. They showed that FLASH RT, compared to CONV, spared fewer immune cells with cardiac and splenic irradiation at 35 Gy/s. Lymphocyte depletion was more severe and sustained with FLASH than CONV for CD3-, CD4-, CD8-, and CD19-positive immune cells. Additionally, FLASH was more potent in causing gastrointestinal mucosal toxicity than CONV. FLASH-irradiated mice died within 7 days compared to 15 days with CONV. 

In a different study, microbeam RT (MRT) of the whole and partial body of mice and its associated effects was assessed. These data also did not show a normal tissue sparing effect with FLASH [54]. In this specific study, three irradiation modalities were compared: MRT (276–319 Gy/s), synchrotron broad beam radiation therapy (37–41 Gy/s), and conventional radiation therapy (0.05–0.06 Gy/s). Pulmonary and gastrointestinal toxicity and long-term growth impairment were seen when mice were irradiated with a FLASH modality. After whole body irradiation of mice, all radiation techniques resulted in weight-loss. Following abdominal irradiation only, all mice showed subnormal weight, with abnormal mucosal absorption. Mice that received microbeam cranial irradiation experienced neurological toxicities (ataxia and loss of balance), and all groups had sub-normal weight gain compared to the non-irradiated controls. The MRT-irradiated mice experienced severe neurological toxicities, severe clinical symptoms (hunched posture, lack of grooming, and poor body condition), and significant weight loss. Each radiation set-up showed signs of inflammation and long-term pulmonary destruction.

### 3.6. Zebrafish

Zebrafish have also been used as models to investigate the FLASH effect. Beyreuther et al. [55] tested the proton FLASH effect versus proton CONV on zebrafish embryos. Zebrafish were irradiated with either 5 Gy/min CONV or 100 Gy/s FLASH. The dose-dependent embryonic survival data showed a time-dependent decrease for doses >15 Gy. No difference was obtained for dose-dependent malformations, except for pericardial edema, which was significantly reduced after proton FLASH irradiation. However, the overall dose response was not affected. Another study reported that zebrafish irradiated with FLASH showed fewer alterations in body length than after CONV [4,5].

**Table 2:** Summary of in vivo studies investigating the FLASH effect. Acute and late effects, assessment on tumor control, and corresponding FLASH effect for each study are described, compared to CONV RT.

Animal Model (Area/Tumor)	Mean Dose Rate (Gy/s)	Radiation Dose (Gy)	FLASH Source	FLASH-Induced		Tumor Control	FLASH Effect	Reference
				Acute Effects	Late Effects			
*Murine models*								
Mice (brain)	35	NS	Electrons	Increased lymphocyte depletion	Worse overall survival	NS	No	[53]
Mice (spleen)				Gastrointestinal mucosal toxicity				
Mice (partial body)	37–41	NS	Photons	Gastrointestinal toxicity Low body weight Neurological toxicity Clinical symptoms Inflammation	Growth impairment Pulmonary destruction	NS	No	[54]
Mice (xenograft human lungs)	40	8	Electrons	Protection from apoptosis	Decreased lung fibrosis	Equal	Yes	[18]
Mice (lung carcinoma)	40	18	Protons	Increased lymphocyte recruitment	NS	Improved	Yes	[32]
Mice (brain)	40	8	Electrons	NS	Neurocognitive effects	NS	Yes	[4]
Mice (focal abdomen)	63	12/18	Protons	Less intestinal damage	Decreased intestinal fibrosis	Equal to CONV RT	Yes	[49]
Mice (subcutaneous pancreatic tumor)	63	12/18						
Mice (leg)	65–92	31.2–53.5	Protons	Skin toxicity	NS	NS	Yes	[56]
Mice (subcutaneous glioblastoma)	66	8 Gy × 2	Electrons	NS	NS	Yes	Yes	[48]
Mice (intracranial glioblastoma)	74	12.5 Gy × 2	Electrons	NS	NS	Yes	Yes	
Mice (whole abdomen)	94	15	Protons	Increased proliferating cells per crypt	Reduced intestinal fibrosis	NS	Yes	[49]
Mice (lymphoblastic leukemia and normal hematopoiesis)	200	4	Electrons	NS	Decrease in leukemic cells Difference in genetic factors Preservation of hematopoietic/ progenitor cells	Improved	Yes	[52]
Mice (whole brain)	200–300	30	Electrons	No loss of dendrites Decreased neuroinflammation	Protection from neurocognitive effects	NS	Yes	[14]
Mice (ovarian cancer)	216	14–16	Electrons	Body weight Hematopoietic toxicity DNA damage Apoptosis	Better overall survival Similar mucosal damage Sparing of intestinal function Sparing of epithelial integrity	NS	Yes	[50]
Mice (whole body)	276–319	NS	Photons	Gastrointestinal toxicity Body weight Neurological toxicity Clinical symptoms Inflammation	Growth impairment Pulmonary destruction	NS	No	[54]
Mice (subcutaneous lung carcinoma)	352	15	Electrons	No tumor vascular collapse Increased ROS levels Increased immune cell infiltration	NS	NS	NS	[45]
Mice (orthotopic glioblastoma)	1.9 × 10^6^	3.5 Gy × 4	Electrons	No neurocognitive effects	Tumor control Overall survival	Equal	No	[46]
	2.5 × 10^6^	25 Gy				Yes	Yes	
	3.9 × 10^6^	7 Gy × 2				Equal	Yes	
	5.6 × 10^6^	10 Gy × 3				Equal	Yes	
	5.6 × 10^6^	10				Yes	Yes	
	7.8 × 10^6^	14		Impaired neurocognitive effects		Equal	No	
Juvenile mice (whole brain)	4.4 × 10^6^	8	Electrons	Attenuated memory-impaired functions Preservation of growth hormones	Recovered impaired memory updating Preserved neurogenesis Minimized anxiety-like behaviors	NS	Yes	[47]
Mice (whole abdomen)	2–6 × 10^6^	7.5–20	Electrons	Increased crypt survival Reduced change in gut microbiome	NS	NS	Yes	[51]
Mice (whole brain)	NS	10	X-rays	Reduced astrogliosis	Protection from neurocognitive effects	NS	Yes	[13]
Mice (lungs)	NS	NS	Electrons	Less DNA damage Minimized induction of pro-inflammatory genes	Less senescence Decreased fibrosis	NS	Yes	[12]
*Fishes*								
Zebrafish	40	8	Electrons	NS	Neurocognitive effects	NS	Yes	[4]
Zebrafish embryo	100	NS	Protons	NS	No difference in malformation Reduced pericardial edema No difference in survival	NS	Yes	[55]
Zebrafish embryo	177; 287; 2.5 × 10^5^	32	Electrons	Reduced morphological alterations	NS	NS	Yes	[57]
	300	30	Protons					
Zebrafish embryo	1 × 10^5^	26	Electrons	Reduced morphological alterations	NS	NS	NS	[58]
*Large animals*								
Mini pigs	150	31	Electrons	Depilation	Erythema Ulceration Hyperkeratosis Skin contracture	NS		[59]
Mini pig (skin)	160	31–41	Electrons	Preserved hair follicles	Decreased fibrosis No permanent late toxicities	NS	Yes	[16]
Cat (nasal planum)	300–400	41	Electrons	Permanent depilation				
Cat (nasal planum)	1500	30	Electrons	1 observation: Moist desquamation	Mucosal breakdown Bone necrosis	NS	Yes	[59]
Dog (leg)	61–128	4–12	Protons	Decreased TGF-β levels	NS	Yes	Yes	[60]
Dog	400–500	8 or 12	Electrons	Alopecia Desquamation Leukotricia Mild erythema	NS	Yes	Yes	[61]

NS: not studied; TGF-β: transforming growth factor-beta.

In the presence of ROS-scavenging agents, however, CONV-treated zebrafish embryos exhibited less morphological alterations, while no difference could be observed in the FLASH-treated group. In order to investigate determinants in the FLASH effect in zebrafish, Karsch et al. included FLASH dose rates using electrons and protons resembling isochronous cyclotrons, synchrocyclotrons, and synchrotrons. Maximal sparing effects were dependent on the mean dose rate, but also the radiation time [57], as seen in the mice study by Ruan et al. [51]. FLASH experiments using high pulse dose rate on the zebrafish embryo model demonstrated a protective effect relative to the controls [57]. They observed slightly less reduction in embryo length as well as a reduction of about 20–25% of embryos with spinal curvature and pericardial edema. Furthermore, low partial oxygen levels also appeared to have a stronger FLASH effect compared to high partial oxygen levels [58].

### 3.7. Larger Animals

Larger animals such as the mini pig, cat, and dog cancer patients have also been assessed using FLASH and CONV RT [16,59,60,61]. Following irradiation, early effects on skin toxicity as well as late fibrosis were evaluated in the mini pig. The study demonstrated that after up to 31 Gy FLASH, regrowth of hair was first observed at 14 weeks compared to 22 Gy and 24 weeks for CONV. Late skin toxicities were reduced at 32 weeks post-irradiation. Hair follicles were preserved following FLASH RT but destroyed after CONV RT. FLASH-irradiated skin retained the expression of CD34, indicating minimal impact on epidermal stem cells. Additionally, fibronecrotic scabs fell off around 42 weeks post-FLASH RT. Their data showed a dose-modifying factor of at least 20% for single fraction treatment in favor of FLASH for the protection of normal tissue and prevention of fibrosis as endpoints. However, late skin toxicity in a subsequent study with mini pigs was observed with increasing volume, including permanent hyperkeratosis and skin contracture [59].

Cats with squamous cell carcinoma of the nasal planum were treated with FLASH RT for evaluation of late toxicities, antitumor efficacy, and overall survival [16]. With a median follow-up of 18 months, all six cats revealed permanent depilation, which was restricted to the irradiated field. No other permanent late toxicities were observed. All six cat patients were assessed during follow-up to evaluate tumor control and overall survival. After 6 months, a complete response was observed for all cats. At 16 months, five of the six cats were still disease free, and after 18 months, three of the six cats were still disease free [16]. Furthermore, comparison between CONV and FLASH RT in cats showed that 30 Gy at a mean dose rate of 1500 Gy/s led to osteoradionecrosis in three out of seven cats in the FLASH RT arm, resulting in a preliminary termination of the trial [59]. This potentially important toxicity signal requires further detailed evaluation and explanation.

Canine cancer patients with either spontaneous superficial tumors or microscopic disease were irradiated with 15–35 Gy FLASH. Eleven out of thirteen irradiated tumors showed partial or complete response, or stable disease. Grade 1 adverse effects such as mild local alopecia, leukotricia, dry desquamation, and mild erythema or swelling were observed after 3 to 6 months. One canine patient developed a grade 3 skin adverse event. The results indicate FLASH treatment of oropharyngeal tumors to be feasible [61].

## 4. Towards the Clinic

### 4.1. The First Human Study

The first patient treated with FLASH had a multi-resistant CD30+ T cell cutaneous lymphoma and received electron beam FLASH using a single dose of 15 Gy in 90 ms [17]. Redness of the skin was observed between days 10 and 44, grade 1 asymptomatic mild epithelitis after 3 weeks, and grade 1 edema between days 12 and 24. The tumor shrunk at 10 days, and at 36 days, tumor response was complete. The study demonstrated the technical feasibility using FLASH-RT in a human patient with encouraging results.

The FAST-01 clinical trial (NTC04592887) is currently active to assess the feasibility of proton FLASH RT for the treatment of painful bone metastases [62]. Pain response and adverse side-effects will be reported, and the workflow feasibility of the treatment will be evaluated. Another phase I clinical trial (NCT04986696) is now recruiting patients with skin melanoma metastases [62]. The trial will evaluate single dose escalation using the Mobetron electron-beam FLASH. More clinical trials using FLASH RT are planned, including breast cancer treatment using intraoperative radiotherapy [62].

### 4.2. Devices for Clinical FLASH RT

Substantial progress has been made regarding the technical development of FLASH-RT systems and the physics of ultra-high dose rate irradiation. For a more detailed discussion we refer to a number of recent reviews [63,64,65,66,67]. Here, we highlight a few specific examples, with an emphasis on devices designed with clinical use in mind. While most preclinical FLASH studies have been performed using electrons (Table 2), the results of such experiments are being extrapolated to photons, protons, and other types of radiation (e.g., carbon ions), for which there is emerging preclinical data [46,68,69]. The low penetration depth of electron beams is adequate for preclinical studies and the treatment of superficial tumors/surfaces in the clinic (e.g., skin metastases, intra-operative treatments), and machines are being developed for these clinical indications, including FLASHKNiFE [70,71], IntraOp Mobetron [72], and modified NOVAC7 [73]. Electron FLASH has already been used in the clinic, and trials have been initiated. These electron devices are insufficient for the external targeting of deeper tumors. For this indication, photon FLASH, which is currently not clinically available, and proton FLASH, which has already been used in a clinical study, are being developed. PHASER (pluridirectional high-energy agile scanning electronic radiotherapy) is being developed for the near-instantaneous delivery of multi-beam photon FLASH [74]. Another development includes the high-energy X-ray PARTER (platform for advanced radiotherapy research) system [68]. Proton FLASH has already entered clinical trials using transmission (as opposed to Bragg-peak) beams, and Varian Medical Systems, which is sponsoring the FAST-01 and FAST-02 trials, received an Investigational Device Exemption (IDE) from the U.S. Food and Drug Administration (FDA) to use a modified ProBeam proton system and Eclipse treatment planning system for clinical trial use. The development of FLASH RT systems suitable for routine clinical practice is still a great multidisciplinary challenge, with most promising perspectives for the cancer patient.

### 4.3. Clinical Translation

Promising data from in vitro and in vivo studies and treatment of the first human patient show the potential for clinical translation. Consequently, the first clinical trials are emerging. Despite extensive radiobiology research into the FLASH effect, it is not yet fully understood (Table 1). However, there are several findings that support translation of the preclinical data into the clinic. In addition to in vivo studies on small rodents, preclinical data have also been obtained using large experimental animals, such as cats, dogs, and a mini-pig [16,60,61]. Large animal data reflect the human situation even better than small animal data. Furthermore, the preclinical data from both small and large animal studies are quite consistent. Murine studies on normal tissues show significant sparing following irradiation with FLASH compared to CONV (Table 2). However, it is important to note that (1) the size of the normal tissue-sparing effect is different between different tissue types, and (2) so far, studies are restricted to a limited number of normal tissue types. In addition, the radiation-induced immune response may also be altered in different tumor types [75,76]. Tumor control was maintained in various mouse tumor models of breast, lung, head and neck, ovarian, and brain cancer; sarcomas; and a fibrosarcoma using FLASH RT (Table 2). Orthotopic models are expected to feature prominently in future work as understanding of the TME increases, including its role in tumor progression. Taken together, much of the available preclinical data support a differential effect between tumor and normal tissue for FLASH RT. Therefore, the data hint that dose escalation with FLASH may be possible, aimed at enhancing tumor control at isotoxic normal tissue effects. At the same time, it must be noted that there are some inconsistencies and limitations in the preclinical studies [53,54]. A proportion of the studies contain significant limitations, such as single subjects and lack of a CONV control group. The positive studies suggest dose modification with a factor of 1.2–1.4 and a FLASH effect occurring at high single doses exceeding 10 Gy. More information about the potential for FLASH RT effects at lower doses and in fractionated treatments is needed. In view of these limitations, initial safety studies, eventually followed, if appropriate, by randomized controlled trials with a FLASH and CONV arm will be required to definitively establish the likely therapeutic benefit of FLASH RT.

## 5. Conclusions

Many in vivo studies using ultra-high dose rate radiation provide supporting evidence for the clinical translation of FLASH RT. However, there are some inconsistencies regarding the FLASH-sparing effect on normal tissues, which might be attributed to the specific experimental conditions and irradiation protocols. Data are available for different normal tissues (Table 2), but long-term data on critical, radiation dose-limiting, late-responding normal tissues such as the kidney and the spinal cord are not yet available. Tumor control studies on several tumor types in small and large animal models demonstrate FLASH-RT to be iso-effective to CONV. Orthotopic tumor models, i.e., with the tumor in its natural micromilieu, and studies incorporating clinically relevant dose-fractionation schemes are expected to feature more heavily in future work. Future modulation studies of the oxygen effect, the ROS recovery rate, the DNA damage response, and the immune reaction might further contribute to understanding of the underlying biological mechanisms of the ultra-high dose rate effect. It is anticipated that future work may also focus on identifying molecular targets to further enhance the FLASH effect between normal and malignant tissue, for example, via radiosensitization and immune-modulation strategies.

## Figures and Tables

**Table 1:** Overview of intrinsic factors that potentially influence the differential effect between FLASH and CONV, both in normal tissues and tumors.

Factor	Normal Tissue	Tumor	Normal and Tumor
Oxygen depletion hypothesis			
Oxygen [23,24]	Rapid oxygen depletion	Small change in oxygen	-
ROS [4,25]	Reduction of ROS	No change of ROS	-
Oxygen to hydroperoxide conversion [25]	High removal of hydroperoxides	Slow removal of hydroperoxides	-
Capillary oxygen Tension [24]	Higher	Lower	-
DNA damage hypothesis			
Yields of DNA damage [26]	Smaller amounts of DSBs	Higher amount of DSBs	-
Pattern of DNA Damage [27]	Higher amount of clustered DNA damage will lead to activation of different factors (DNA repair, immune system)	Lower amount of clustered DNA damage will lead to activation of different factors (DNA repair, immune system)	-
DNA damage repair pathways [28,29]	Unknown pathway, decreasing ROS and DNA damage	PARP-TGF-β pathway	-
Factors induced by DNA damage [30,31]	-	-	Initiation of cGAS-STING pathway is different between tumor and healthy tissue
Immune hypothesis			
TGF-β and other immune factors [18,26]	Reduction of TGF-β	Induction of TGF-β	-
Immune cells and microenvironment [32]	-	Increase of T-lymphocytes into the tumor microenvironment	-
Immunogenic cell death [33]	-	-	Effects of FLASH on immunogenic cell death remain unclear

DSBs: double-stranded breaks; PARP: poly (ADP-ribose) polymerase; ROS: reactive oxygen species, TGF-β: transforming growth factor-beta.

## Data Availability

Not applicable.
